# NMR Study on Laccase Polymerization of Kraft Lignin Using Different Enzymes Source

**DOI:** 10.3390/ijms24032359

**Published:** 2023-01-25

**Authors:** David Ibarra, Luisa García-Fuentevilla, Gabriela Domínguez, Raquel Martín-Sampedro, Manuel Hernández, María E. Arias, José I. Santos, María E. Eugenio

**Affiliations:** 1 Forest Sciences Institute (ICIFOR-INIA), CSIC, Ctra. de la Coruña Km 7.5, 28040 Madrid, Spain; 2 Department of Biomedicine and Biotechnology, University of Alcalá, 28805 Alcalá de Henares, Spain; 3 General Services of Research SGIKER, University of the Basque Country (UPV/EHU), Edificio Joxe Mari Korta Avda. Tolosa 72, 20018 Donostia-San Sebastian, Spain

**Keywords:** eucalypt, Kraft lignin, laccase, NMR characterization, polymerization

## Abstract

The usage of laccases is a sustainable and environmentally friendly approach to modifying the Kraft lignin structure for use in certain applications. However, the inherent structure of Kraft lignin, as well as that resulting from laccase modification, still presents challenges for fundamental comprehension and successful lignin valorization. In this study, bacterial and fungal laccases were employed to modify eucalypt Kraft lignin. To evaluate the type and range of the chemical and structural changes of laccase-treated lignins, different NMR techniques, including solution ^1^H and 2D NMR (heteronuclear single quantum correlation (HSQC)), and solid-state ^13^C NMR, were applied. Size exclusion chromatography and infrared spectroscopy were also used. Interestingly, HSQC analysis showed substantial changes in the oxygenated aliphatic region of lignins, showing an almost complete absence of signals corresponding to side-chains due to laccase depolymerization. Simultaneously, a significant loss of aromatic signals was observed by HSQC and ^1^H NMR, which was attributed to a deprotonation of the lignin benzenic rings due to polymerization/condensation by laccase reactions. Then, condensed structures, such as α-5′, 5-5′, and 4-O-5′, were detected by HSQC and ^13^C NMR, supporting the increment in molecular weight, as well as the phenolic content reduction determined in lignins.

## 1. Introduction

Lignin is the second most abundant biological macromolecule on earth, giving lignocellulose biomass rigidity, water impermeability, and protection against microbial decay and mechanical stress [1]. Currently, lignin is widely generated as a side-stream of the pulping processes used for lignocellulosic biomass fractionation in different industrial sectors. Among the different pulping processes, the Kraft process is the most extended pulping technology, with an average lignin production estimated at 130 million tons per year [2]. The major fraction of this Kraft lignin is simply burned, due to its high calorific value, to produce energy, i.e., heat and power. However, lignin valorization into high added-value chemicals and materials has started to attract significant attention in the last years [3], helping to increase the sustainability and competitiveness of this industrial sector. In addition, Kraft lignin valorization is also expected to contribute to the implementation of the biorefinery and circular bioeconomy concepts in the pulp and paper industry, which aims to maximize the usage and value of all raw materials, products, and side-streams.

The valorization of Kraft lignins largely depends on tailoring their physico-chemical properties for the different applications intended, including molecular weight distribution and presence of reactive groups. Different chemical treatments, such as oxidative modifications, can be used to change these Kraft lignin properties [4]. However, the harsh application conditions, as well as the economic costs of some of them, limit their applicability. As environmentally friendly alternatives, oxidative enzymes, that is, laccases and peroxidases, involved in lignin biosynthesis in nature, can accomplish this oxidative modification [5]. Laccases (benzenediol/oxygen oxidoreductases and EC 1.10.3.2) are multicopper-containing oxidases with phenoloxidase activity, which enables them to catalyze the oxidation of a broad diversity of phenolic and non-phenolic molecules, using oxygen as the final electron acceptor and releasing water as a by-product [6]. This oxidative capacity, together with the low requirements, and the ability to catalyze polymerization reactions, make laccases suitable for the modification of lignin structure to produce the appropriate lignin for each potential application. Then, the laccase polymerization of lignin has been recently reported in the manufacture of new lignin-based products. They include nanocomposite materials with nanocellulose [7], green adhesives for wood-based panels [8], biolubricants [9], pesticide delivery systems [10], fertilizer-controlled release systems [11], paints and coatings, thermosets, and carbon fiber precursors [12]. In most of these studies, fungal enzymes are usually employed [8,10,11,13,14,15,16,17,18]. Only recently, bacterial enzymes have also received interest for this purpose [7,19,20].

The inherent structure of Kraft lignin, as well as that resulting from laccase polymerization, still present challenges for fundamental comprehension and successful valorization of lignin. Then, some of the most powerful tools for the structural characterization of lignin, such as multidimensional nuclear magnetic resonance (NMR) spectroscopy, are being applied for this purpose [7,8,14,18]. In the present study, different NMR techniques, including solution ^1^H NMR and 2D NMR (based on heteronuclear single quantum correlation (HSQC)), and solid-state CP/MAS (cross polarization/magic angle spinning) ^13^C NMR, were used to investigate the type and range of the chemical and structural changes of eucalypt Kraft lignin, produced by two different laccases of bacterial and fungal origin. In addition, size exclusion chromatography (SEC) and Fourier transform infrared (FTIR) spectroscopy were also employed.

## 2. Results and Discussion

In this study, bacterial and fungal laccases, namely, from *Streptomyces ipomoeae* (SiLA) and *Myceliophtora thermophila* (MtL), respectively, were used to polymerize Kraft lignin from *Eucalyptus globulus*. These enzymes show a variety of properties, such as stability at alkaline pH and high temperature, that make them appropriate for lignin polymerization, as it has been previously shown [9,16]. Subsequently, different NMR techniques, including ^1^H NMR, 2D NMR (HSQC), and CP/MAS ^13^C NMR, were used to investigate the Kraft lignin, as well as the type and range of the chemical and structural changes produced by laccase enzymes.

### 2.1. Phenolic Content

As expected, similar to trends in previous studies [21,22], a high phenolic content was observed in untreated lignin as a result of its depolymerization during alkaline Kraft pulping conditions (Table 1), which help to solubilize lignin in black liquor [22,23]. The low redox potential reported for both laccases (0.450 mV) prevents them from oxidizing the non-phenolic lignin [24,25]. Therefore, the initiation of lignin oxidation by both laccases is believed to occur at these phenolic hydroxyl groups, yielding resonance-stabilized phenoxyl radicals via a single electron transfer process [26]. In this sense, both SiLA and MtL laccases showed the capacity to decrease the phenolic content of the original Kraft lignin, observing a higher reduction of total phenolic content when the bacterial laccase was used compared to the fungal laccase (Table 1). Then, SiLA laccase produced a phenolic content decrease of 64.3% using 40 IU/g of lignin for 90 min (SiLA-KL1), whereas a phenolic content reduction of 32.9% was produced by MtL laccase at similar conditions (MtL-K1). 

The phenolic content reduction of different lignin side-streams has been described either by bacterial or fungal laccases. Wang et al. [7] reported the ability of a commercial bacterial laccase (Metzyme^®^, 1 IU/mg lignin for 6 h) to oxidize different lignin fractions derived from birch and spruce alkali lignins, observing an important total phenolic content reduction (between 30% and 70%). Mayr et al. [20] also showed the potential of the bacterial laccase CotA (spore coating protein A, 1 IU/mg lignin for 6 h) for Kraft lignin oxidation, describing a phenolic content reduction between 30% and 65%, depending on the softwood and hardwood Kraft lignin sources. Regarding fungal laccases, Prasetyo et al. [18] reported a phenolic content reduction between 39% and 47% in lignosulfonates when treated with the fungal laccases from *Trametes villosa* and *Trametes hirsuta* (90 IU/g lignin for 83 h), respectively. Finally, MtL laccase (1333 IU/g lignin for 2 h) produced a phenolic content reduction of eucalypt Kraft lignin around 66% [8].

Increasing the laccase dosage to 100 IU/g of lignin and the incubation time to 240 min resulted in a mild further phenolic content decrease of the resulting laccase-treated lignins. Then, SiLA produced a phenolic content decrease of 65.7% (SiLA-KL2), whereas a phenolic content reduction of 38.6% was achieved by MtL laccase (MtL-KL2). In a previous work by Mayr et al. [20], a higher phenolic content decrease throughout the 6 h reaction was reported when the bacterial laccase CotA was used in higher doses to polymerize softwood and hardwood Kraft lignins. Huber et al. [27] evaluated the effect of MtL laccase dosage and reaction time on the polymerization of Kraft lignin and lignosulfonates. They determined that when using 50 mg of laccase, the phenolic content decrease was 24.3% for the Kraft lignin and 30.9% for lignosulfonates. When the laccase concentration was increased 2-fold, a further decrease of the phenolic content for both lignins (30.8% for Kraft lignin and 52% for lignosulfonates) was observed. Moreover, a longer reaction time resulted in higher reductions of the phenolic content, observing the maximum reduction for both types of lignins and enzyme concentrations at 24 h. 

### 2.2. Size Exclusion Chromatography 

Weight-average (Mw) and number-average (Mn) molecular weights, as well as polydispersity (Mw/Mn) values, showed in Table 1 were obtained from the molecular weight distributions of untreated and laccase-treated lignins (Figure S1). The low molecular weight value of untreated lignin (3.5 KDa) clearly revealed the high degradation of the lignin macromolecule during the Kraft pulping process, which is related to a broad cleavage of β-O-4′ linkages [22,28], the dominant aryl ether bond in native lignin [29]. After enzymatic treatment, both SiLA and MtL laccases produced increments in the Mw values of treated Kraft lignins using 40 IU/g of lignin for 90 min. Then, SiLA laccase produced a 1.8-fold increase in the Mw value (SiLA-KL1), whereas a 1.7-fold increase was observed when MtL laccase was used (MtL-KL1) (Table 1). The phenoxyl radicals generated by laccase oxidation undergo resonance stabilization, forming different mesomeric forms that couple in many possibilities, yielding inter-unit linkages, such as phenyl ether-carbon and carbon-carbon linkages [18], and consequently, resulting in the observed increments in the Mw values. Together with Mw increase, polydispersity was also augmented (Table 1). This is normally because of the nature of the non-selective radical–radical polymerization caused by laccase oxidation of phenolic end groups in Kraft lignin. The coupling reactions binding lignin end groups to each other occur spontaneously, with poor or no control, and because the radicals that initiate the polymerization are available, the reaction is propagated, with subsequent polydispersity increase [30].

The molecular weight increment of different lignin side-streams has been described either by bacterial or fungal laccases. Wang et al. [7] described a 2.9-fold increase in the molecular weight of the alkali spruce lignin treated with a bacterial commercial laccase (Metzyme^®^). Mayr et al. [20] reported increments in molecular weight of 6.0-fold for softwood and 19.2-fold for hardwood Kraft lignins when they were treated with the bacterial CotA laccase. Fiţigău et al. [13] showed increments in molecular weights (between 3.0-fold and 5.0-fold) of different technical lignins, e.g., hardwood, softwood, and grass lignins subjected to alkaline, soda, and organosolv processes, when they were treated with a laccase from *T. versicolor* (0.75 IU/mg lignin for 24 h). Huber et al. [27] achieved increases in molecular weight of 12.0-fold for enzymatic polymerization of lignosulfonates, and only a 1.4-fold increase for Kraft lignin, using the fungal MtL laccase. However, with this MtL laccase, Gouveia et al. [8] showed a strong increase (17.0-fold) in the average molecular weight of laccase-treated eucalypt Kraft lignin, probably due to the use of different lignin sources in the laccase polymerization reactions.

An increase in laccase dosage (100 IU/g of lignin) and reaction time (240 min) also led to a mild further increase in the Mw values. Then, SiLA laccase produced a 2.4-fold increase of the molecular weight (SiLA-KL2), whereas a 2.2-fold increase was observed when MtL laccase was used (MtL-KL2). Previously, Mayr et al. [20] also achieved higher increases in the molecular weight values of softwood and hardwood Kraft lignins at longer reaction times using a bacterial CotA laccase, observing the maximum values at 6 h and 9 h, respectively. Huber et al. [27] determined that using 50 mg of MtL laccase, a 4.0-fold and 1.7-fold molecular weight increment was achieved for lignosulfonates and Kraft lignin, respectively. The results using 100 mg of laccase showed a further increase in the average molecular weight of lignosulfonates (increase of 12.0-fold) compared to the control, whereas for Kraft lignin, the increment was lower (increase of 1.4-fold). In addition, higher molecular weights were achieved at longer reaction time, resulting in the maximum increments at 24 h. Areskogh et al. [30] also evaluated the fungal MtL laccase to polymerize lignosulfonates. They observed that no important increments in the molecular weight of lignosulfonates was produced at a low MtL enzyme dosage (50 IU/g lignin). However, the molecular weight was increased by augmenting the enzyme concentration (500 IU/g lignin). Finally, Magina et al. [31] described increments in the molecular weight of eucalypt lignosulfonates at higher MtL dosages (0–500 IU/g) and incubation times (0–90 min), achieving an 11-fold increase at the highest laccase dosage and reaction time.

### 2.3. FTIR Characterization 

Figure S2 shows the FTIR spectra of untreated and laccase-treated Kraft lignins. The FTIR spectrum of untreated lignin (Figure S2a) displayed the typical bands of hardwood lignins [21,32], with bands attributed to syringyl (S) and guaiacyl (G) units. Then, bands at 1315 cm^−1^ (aromatic ring breathing in S and G condensed units), 1270 cm^−1^ (aromatic ring breathing with C=O stretching in G units), 1220 cm^−1^ (aromatic ring breathing with C–C, C–O, and C=O stretching in G units), 1115 cm^−1^ (C–H bond deformation in S units), 1025 cm^−1^ (C–H bond deformation in G units), and 820 cm^−1^ (C–H out of plane deformation in S units) were observed. Moreover, the characteristic bands associated to the lignin aromatic skeleton (1610, 1515, and 1415 cm^−1^) and to the C−H asymmetric vibrations and deformation (1455 cm^−1^) were also seen.

According to the composition of untreated lignin (Section 3.1), several bands at 1155, 1115, and 1025 cm^−1^ (C–O asymmetric vibration, C–OH skeletal vibration, and C–O stretching vibration, respectively) were tentatively endorsed to cellulose and hemicelluloses. On the other hand, a shoulder at 1715 cm^−1^ was attributed to the carbonyl groups of hemicelluloses. Nevertheless, this band can be also associated to the unconjugated C=O stretching resulting from lignin oxidation [33]. 

Due to the lignin oxidation produced by the SiLA and MtL laccases, their corresponding FTIR spectra showed a noticeable intensity increment at 1715 cm^−1^ (C=O stretching for unconjugated linkages) and at 1650 cm^−1^ (C=O stretching for conjugated linkages) (Figure S2b–e), especially when the bacterial laccase was used (Figure S2b,d). This observation has been previously described in different studies. Zhu et al. [34] reported the oxidation of the hydroxyl groups on the Cα of the lignin (alkaline lignin) side-chain when it was treated with a bacterial laccase from *Bacillus ligniniphilus*. Similarly, Areskogh et al. [35] also described the formation of carbonyl groups in the side-chain of lignosulfonates during polymerization reactions with MtL laccase, and Fiţigău et al. [13] of different technical lignins subjected to *T. versicolor* laccase treatment. However, Aracri et al. [36] attributed the presence of carbonyl groups in FTIR spectra to the formation of quinone structures in alkaline lignins from annual plants treated with MtL laccase. 

On the other hand, laccase-treated lignins did not show modifications in the bands associated to the lignin aromatic skeleton, in the same way as that observed by Gouveia et at. [16] when MtL laccase was used to polymerize eucalypt Kraft lignin, and Areskogh et al. [35] using the same enzyme to polymerize lignosulfonates.

### 2.4. NMR Characterization 

#### 2.4.1. Two-Dimensional NMR (Heteronuclear Single Quantum Correlation (HSQC))

**Figure 1:**
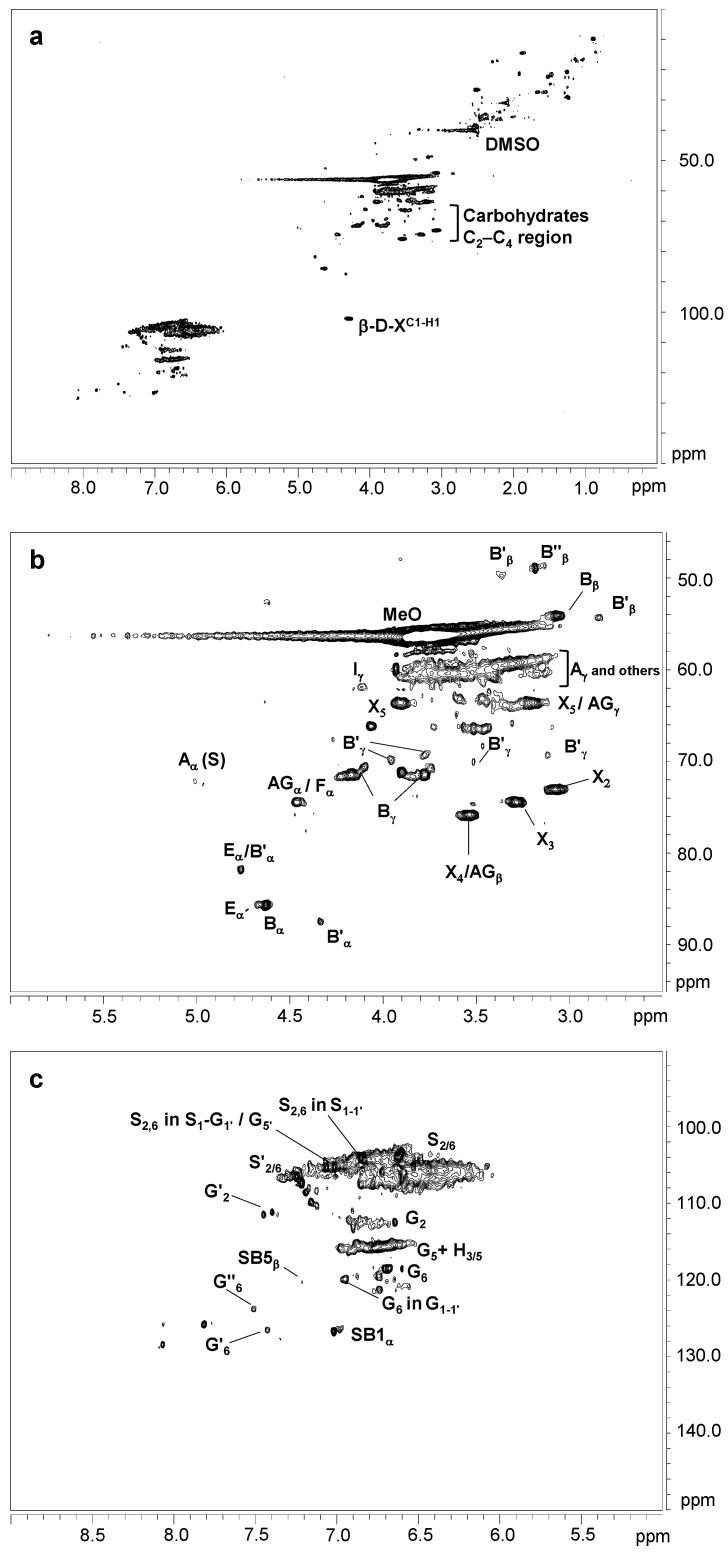
HSQC 2D-NMR spectra of untreated Kraft lignin. (**a**) whole spectrum, δ_C_/δ_H_ 0.0–150.0/0.0–9.0; (**b**) aliphatic oxygenated region, δ_C_/δ_H_ 45.0–95.0/2.5–6.0 ppm; (**c**) aromatic region, δ_C_/δ_H_ 90.0–150.0/5.0–9.0 ppm.

**Figure 2:**
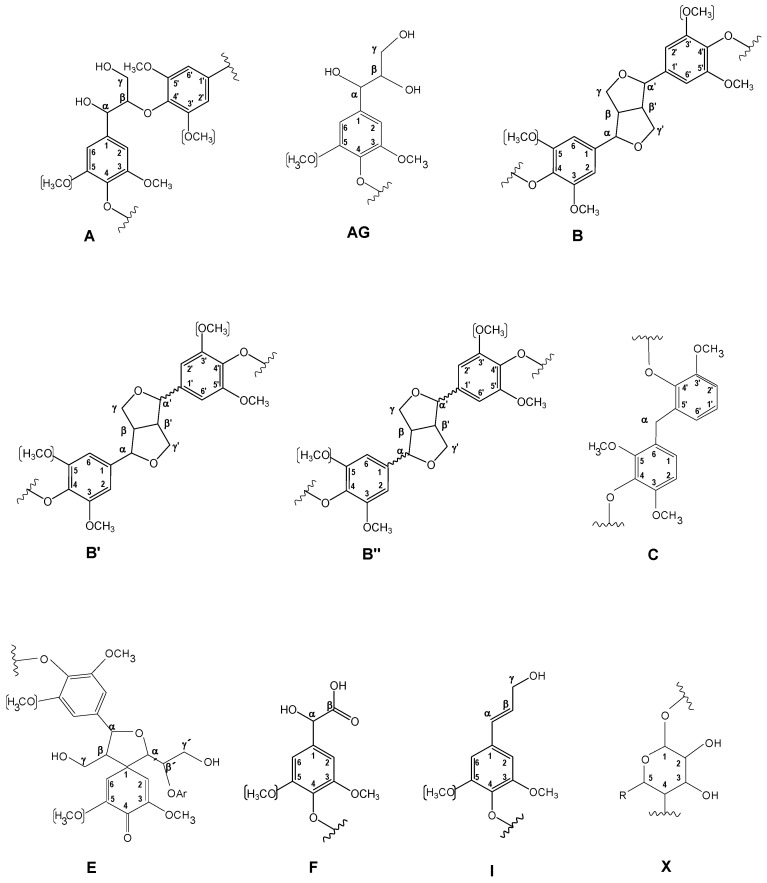
Main lignin and carbohydrate substructures identified in aliphatic oxygenated region of untreated and laccase-treated Kraft lignins with MtL and SiLA laccases. **A**, β-O-4′ alkyl-aryl ether; **AG**, aryl-glycerol; **B**, resinols; **B′**, epiresinols; **B′′**, diaresinol; **C**, α-5′; **E**, spirodienones; **F**, Ar–CHOH–COOH; **I**, cinnamyl alcohol end-groups; **X**, xylopyranose (R, OH).

**Figure 3:**
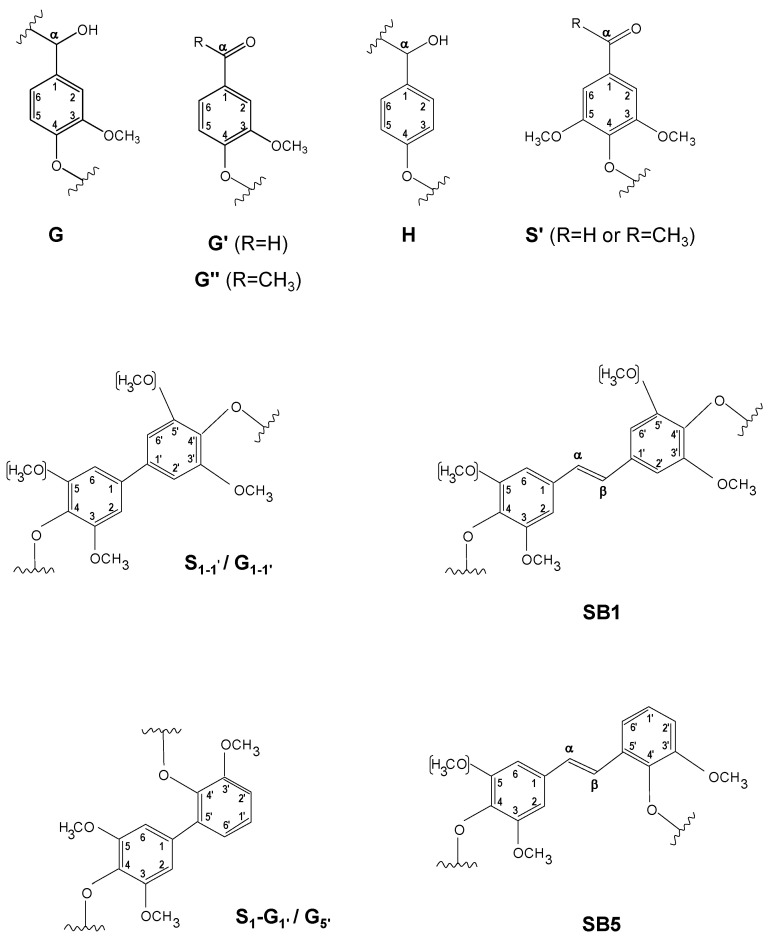
Main lignin substructures identified in aromatic region of untreated and laccase-treated Kraft lignins with MtL and SiLA laccases. **G**, guaiacyl unit; **G′**, vanillin; **G″**, acetovanillona; **H**, *p*-hydroxyphenyl unit; **S′**, syringaldehyde (R=H) or acetosyringone (R=CH_3_); **S_1__–1′_**, 3,5-tetramethoxy-para-diphenol; **G_1__–1__′_**, 3-dimethoxy-para-diphenol; **S_1_-G_1__′_/G_5__′_**; **SB1**, stilbene-β-1′; **SB5**, stilbene-β-5′.

Figure 1 displays the HSQC spectra of untreated eucalypt Kraft lignin, including the whole spectrum (δ_C_/δ_H_ 0.0–150.0/0.0–9.0) (Figure 1a), the oxygenated aliphatic region (δ_C_/δ_H_ 45.0–95.0/2.5–6.0 ppm) spectrum (Figure 1b), and the aromatic region (δ_C_/δ_H_ 90.0–150.0/5.0–9.0 ppm) spectrum (Figure 1c). The main ^13^C–^1^H lignin correlation signals found in the HSQC spectra are showed in Table S1, being assigned according to those reported by the bibliography [21,37,38,39,40]. Finally, the lignin substructures and carbohydrates identified are showed in Figure 2 and Figure 3. 

The non-oxygenated aliphatic region (around δ_C_/δ_H_ 0.0–50.0/0.0–5.0 ppm) displayed a variety of signals. Some of them could be associated to extractives, while others could be attributed to groups neighboring alkene and oxygen-containing groups, such as ethers, carbonyl, and alcohol, which could originate from lignin degradation [37].

The oxygenated aliphatic region of the untreated Kraft lignin spectrum was highly enriched in signals from β-β′ resinol substructures (C_α_–H_α_ (B_α_), C_β_–H_β_ (B_β_), and the double (B_γ_)) (3.5 linkages per 100 aromatic units). Other signals attributed to β-O-4′ (C_α_–H_α_ for β-O-4′ S lignin units (A_α_) and C_γ_–H_γ_ (A_γ_)) were also observed, although in much lower abundance (0.80 linkages per 100 aromatic units). The native resinol substructures, with C−C linkages, are usually stable to the alkaline conditions of Kraft pulping [23,37]. However, native β-O-4′ substructures are preferentially degraded under alkaline Kraft pulping [22,37], supporting the high phenolic content and low molecular weight described in Section 3.1 and Section 3.2, respectively. Correlation signals for other native substructures, such as spirodienones (C_α_–H_α_ (E_α_) and C_α_′–H_α_′ (E_α_′)) (1.2 linkages per 100 aromatic units) and cinnamyl alcohol end-groups (C_γ_–H_γ_ (I_γ_)) (0.8 linkages per 100 aromatic units), were also recognized. On the other hand, Kraft-derived substructures could also be found in this region. Among them, aryl-glycerol substructure, resulting from the alkaline breakdown of non-phenolic β-aryl ether linkage during Kraft pulping [23], could be hesitantly assigned (C_α_–H_α_ (AG_α_), C_β_–H_β_ (AG_β_), and C_γ_–H_γ_ (AG_γ_)) (2.1 linkages per 100 aromatic units). Overlapping with the C_α_–H_α_ correlation signal of aryl-glycerol, a C_α_–H_α_ correlation signal of lignin terminal structures with a carboxyl group in C_β_ (Ar−CHOH−COOH; F_α_), could also be identified (2.1 linkages per 100 aromatic units). Correlation signals from epiresinols (C_α_–H_α_ (B′_α_) (overlapping with C_α_–H_α_ (E_α_) from spirodienones), C_β_–H_β_ (B′_β_), and C_γ_–H_γ_ (B′_γ_)) and diaresinol (C_β_–H_β_ (B′′_β_)) were also found (0.8 linkages per 100 aromatic units). These diastereomers are derived from the transformation of native resinol substructure during Kraft pulping [40,41]. 

According to the composition of Kraft lignin (Section 3.1), carbohydrate signals, either from hexose or pentose units, were also detected in the oxygenated aliphatic region of the Kraft lignin spectrum. They included correlation signals of the xylan chain for C_2_–H_2_ (X_2_), C_3_–H_3_ (X_3_), C_4_–H_4_ (X_4_), and C_5_–H_5_ (X_5_) (Figure 1b), together with the C-1 cross peak for (1-4) β-D-Xylp of xylan (Figure 1a).

In the same way as FTIR analysis (Section 2.3), the aromatic region of the untreated Kraft lignin spectrum showed the characteristic correlation signals of S (C_2,6_–H_2,6_ (S_2,6_)), G (C_2_–H_2_ (G_2_), C_5_–H_5_ (G_5_), and C_6_–H_6_ (G_6_)), and H lignin units (C_3,5_–H_3,5_ (H_3,5_)) (Figure 3c). The S/G ratio was very high (10.6), reflecting that S lignin units, which predominantly form β-O-4 substructures, are preferentially eliminated from the eucalypt wood during Kraft pulping and are enriched in the black liquors [22]. Signals from lignin oxidation, including oxidized S units (C_2,6_–H_2,6_ (S′_2,6_)) from syringaldehyde or acetosyringone, and oxidized G units (C_2_–H_2_ (G′_2_) and C_6_–H_6_ (G′_6_) and C_6_–H_6_ (G′′_6_)) attributed to vanillin and acetovanillone, could also be observed. On the other hand, correlation signals corresponding to Kraft-derived lignin linkages were also found in the aromatic region. They included signals assigned to β1 stilbene (C_α_–H_α_ (SB1_α_)) (2.2 linkages per 100 aromatic units) and β5 stilbene (C_β_–H_β_ (SB5_β_) (0.1 linkages per 100 aromatic units), both resulting from the degradation of spirodienone and β-5′ phenylcoumaran during the Kraft pulping, respectively [39,40]. Signals for S_2,6_ in S_1-1′_ (3,5-tetramethoxy-para-diphenol), G_2_ and G_6_ in G_1-1′_ (3-dimethoxy-para-diphenol), and S_2,6_ in S_1_-G_1′_/G_5′_ were also tentatively assigned as a result of C_α_-C_1_ cleavage in a retro-aldol reaction, followed by a radical coupling reaction during Kraft pulping [28,38,39].

**Figure 4:**
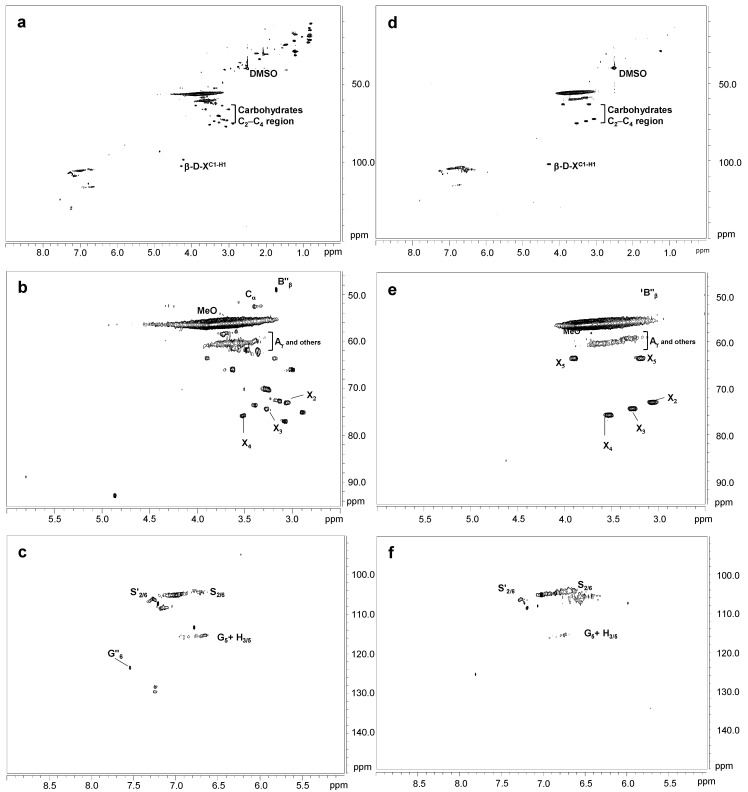
HSQC 2D-NMR spectra of laccase-treated Kraft lignins. Whole spectrum, δ_C_/δ_H_ 0.0–150.0/0.0–9.0, for SiLA-KL1 (**a**) and MtL-KL1 (**d**); aliphatic oxygenated region, δ_C_/δ_H_ 45.0–95.0/2.5–6.0 ppm, for SiLA-KL1 (**b**) and MtL-KL1 (**e**); aromatic region, δ_C_/δ_H_ 90.0–150.0/5.0–9.0 ppm, for SiLA-KL1 (**c**) and MtL-KL1 (**f**).

**Figure 5:**
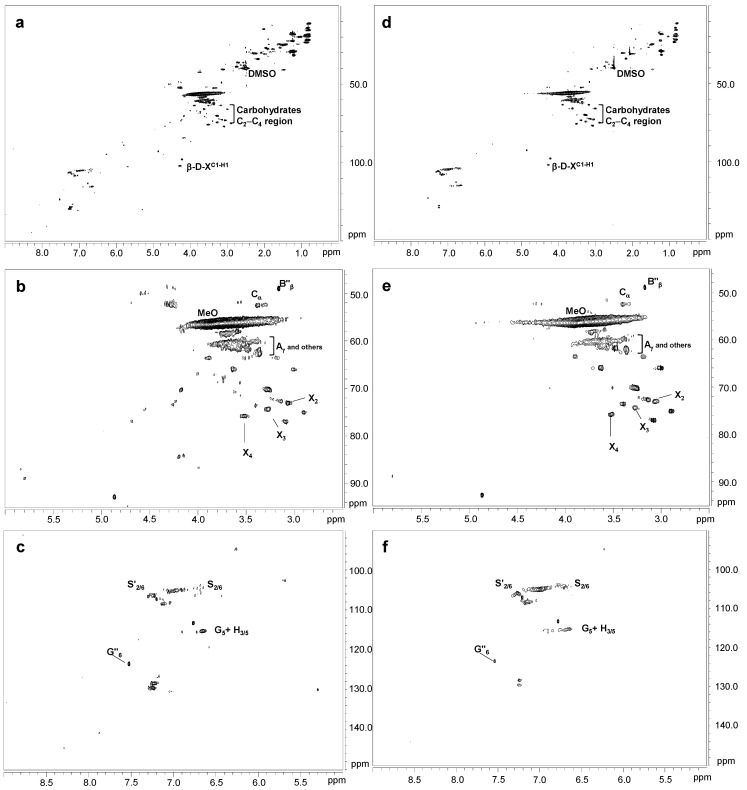
HSQC 2D-NMR spectra of laccase-treated Kraft lignins. Whole spectrum, δ_C_/δ_H_ 0.0–150.0/0.0–9.0, for SiLA-KL2 (**a**) and MtL-KL2 (**d**); aliphatic oxygenated region, δ_C_/δ_H_ 45.0–95.0/2.5–6.0 ppm, for SiLA-KL2 (**b**) and MtL-KL2 (**e**); aromatic region, δ_C_/δ_H_ 90.0–150.0/5.0–9.0 ppm, for SiLA-KL2 (**c**) and MtL-KL2 (**f**).

Figure 4 shows the HSQC spectra corresponding to Kraft lignin treated with 40 IU/g for 90 min of SiLA and MtL laccases (SiLA-KL1 and MtL-KL1, respectively). Compared to the oxygenated aliphatic region of the untreated lignin spectrum (Figure 1a), SiLA-KL1 and MtL-KL1 spectra showed an almost complete absence of signals corresponding to native and Kraft-derived side-chains (Figure 4b and 4e, respectively). Only signals from β-O-4′ (A_γ_) and diaresinol (B″_β_) were still observed, whereas signals from carbohydrates remained practically unaltered by both laccases. Similar observations were displayed for both laccases when the enzyme dosage and reaction time were increased to 100 IU/g and 240 min, respectively (Figure 5b and 5e for SiLA-KL2 and MtL-KL2, respectively). The disappearance of side-chain signals can be explained by the cleavage of these substructures during laccase treatment. In this sense, Wang et al. [7] reported the breakdown of β-aryl ether and β-β′ resinol substructures during the treatment of different alkali lignins with a commercial (MetZyme^®^) bacterial laccase. Sun et al. [42] also described the various bonds’ cleavage, such as β-O-4′, β-1′, β-5′ during modification of alkali lignin with a crude enriched in laccase activity. Prasetyo et al. [18] also described an intensity decrease of signals from β-O-4′ substructures when lignosulfonates were treated with *Trametes villosa* and *Trametes hirsuta* laccases. 

Simultaneously with the disappearance of side-chain signals, a new signal could be found in the aliphatic oxygenated region of the SiLA-KL1 spectrum, at 40 IU/g and 90 min (Figure 4b), and of the SiLA-KL2 and MtL-KL2 spectra, at 100 IU/g and 240 min (Figure 5b and 5e, respectively). This signal was hesitantly attributed to α-5′ (C_α_) condensed structures, which could result from lignin condensation/polymerization reactions by laccases action. In this sense, Wang et al. [7] already reported the formation of α-5′ during the treatment of alkali lignins with the bacterial MetZyme^®^ laccase, proposing different mechanisms for the formation of this condensed structure. On the one hand, the carbocation at C_α_ resulting from the cleavage of side-chains by laccases, mainly from β-β′ resinol substructures, could condense with the electron-rich G_5_ position, among others [7]. On the other hand, quinone methide radicals generated by laccases treatment, from the aryl glycerol, bearing an electron-deficient C_α_, could react with the electron-rich G_5_ position, among others [7,31].

Regarding the aromatic region of the SiLA-KL1 and MtL-KL1 spectra, an important diminution of the aromatic ^13^C–^1^H correlation signals was observed for laccase-treated lignins (Figure 4c and 4f, respectively), with a complete absence at the G_2_ and G_6_ positions. The relative integrals of the total aromatic signals decreased 85.5% for SiLA-KL1 and 78.5% for MtL-KL1. A similar effect was observed for both laccases when the enzyme dosage and reaction time was augmented to 100 IU/g and 240 min, respectively (Figure 5c and 5f for SiLA-KL2 and MtL-KL2, respectively). In this case, the relative integrals of the total aromatic signals decreased 92.0% for SiLA-KL2 and 86.2% for MtL-KL2. The diminution or disappearance of aromatic ^13^C–^1^H correlation signals after laccase treatment have already been described in different studies. Prasetyo et al. [18] reported a complete disappearance when lignosulfonates were treated with *T. villosa* and *T. hirsuta* laccases, whereas a significant reduction was observed by Gillgren et al. [14] when organosolv lignin and lignosulfonates were treated with *C. polyporus* laccase. Wang et al. [7] also described a substantial decrease of ^13^C–^1^H correlation signals, especially S_2,6_ and G_2_ and G_6_ positions, when different alkali lignins were treated with the commercial bacterial laccase MetZyme^®^.

#### 2.4.2. ^1^H NMR and CP/MAS ^13^C NMR

**Figure 6:**
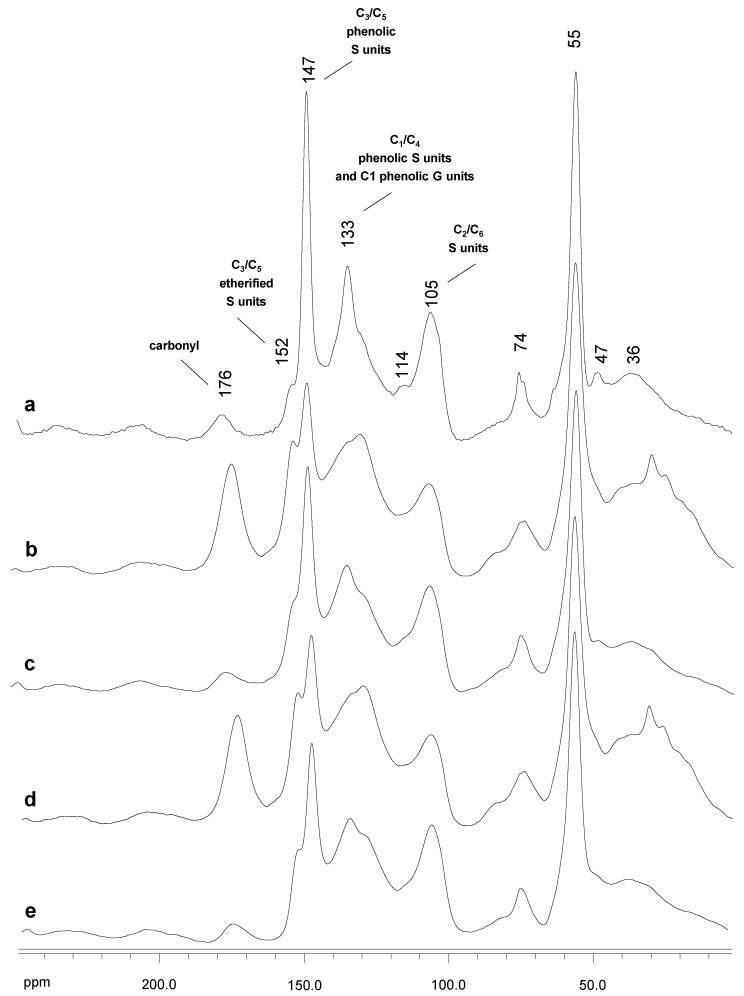
^13^C NMR spectra, δ_C_ 0.0–250.0 ppm, of untreated Kraft lignin (**a**) and the resulting treated lignins with SiLA ((**b**), SiLA-KL1; (**d**), SiLA-KL2) and MtL ((**c**), MtL-KL1; (**e**), MtL-KL2) laccases.

Figure S3 shows the ^1^H NMR of untreated Kraft lignin and the SiLA-KL and MtL-KL samples spectra at the different laccase dosages and reaction times assayed. Compared to the untreated lignin spectrum (Figure S3a), most of the aromatic protons disappeared (7.2–6.2 ppm) after laccase treatment (Figure S3b–e), similar to previous studies [8,18,43,44]. This disappearance of aromatic protons, together with that observed by HSQC experiments, could suggest a substantial modification of the lignin aromatic backbone. However, the ^13^C NMR MtL-KL and SiLA-KL lignins spectra (Figure 6b–e) displayed intensive signals of aromatic carbons, proving that the lignin aromatic skeleton was not degraded by laccase treatment, as previously seen by FTIR analysis (Section 3.3). Therefore, the loss of aromatic signals observed by HSQC and ^1^H NMR after laccase treatment is related to a deprotonation of the lignin benzenic rings as a consequence of polymerization/condensation reactions, such as the formation of α-5′ condensed structures observed by HSQC.

Figure 6 displays the ^13^C NMR of untreated Kraft lignin and the SiLA-KL and MtL-KL samples spectra at the different laccase dosages and reaction times assayed, being the bands assigned according to those described in the literature [28,32,45]. In accordance with the FTIR pattern (Section 2.3), the ^13^C NMR untreated lignin spectrum exhibited a band at δ_C_ 176 ppm endorsed to carbonyl groups and aliphatic COOR from lignin oxidation during alkaline pulping [46]. Nevertheless, hemicelluloses can also contribute to this band [47]. The aromatic region (around δ_C_ 152–95 ppm) showed strong bands at δ_C_ 147 ppm, attributed to C_3_ and C_5_ in S units (phenolic) and C_3_ and C_5_ in G units, and at δ_C_ 133 ppm, associated to C_1_ and C_4_ in phenolic S units and C_1_ in phenolic G units. This high content of phenolic units in eucalypt Kraft lignin observed by ^13^C NMR supported the high phenolic content determined by the Folin–Ciocalteu reagent (Section 2.1). Moreover, a slight shoulder at δ_C_ 152 ppm from non-phenolic units was also detectable. The comparison of the ^13^C NMR-untreated lignin spectrum (Figure 6a) and the laccase-treated lignins spectra (Figure 6b–e) displayed a noticeable increase in the signal at δ_C_ 176 ppm (carbonyl groups), particularly in the SiLA lignin samples (Figure 6b and 6d for SiLA-KL1 and SiLA-KL2, respectively), as previously observed by FTIR analysis (Section 2.3), resulting from the lignin oxidation caused by the laccases action. A similar observation was described by Gouveia et al. [8] when eucalypt Kraft lignin was treated with MtL laccase. Moreover, an abrupt reduction in the intensity of the bands at δ_C_ 147 ppm and 133 ppm was evidenced in the laccase-treated lignins spectra, resulting from the oxidation of the phenolic lignin units by the laccases treatment, which supports the phenolic content decrease observed in Section 3.1. Simultaneously, the intensity of the bands at δ_C_ 152 ppm and at δ_C_ 128 ppm was also visible, especially in the SiLA lignin samples (Figure 6b and 6d for SiLA-KL1 and SiLA-KL2, respectively). These signals have been correlated with the formation of condensed structures by laccase action. Santos et al. [48] endorsed the signal at δ_C_ 152 ppm to the C_3_ in 5-5′ or C_3_ (and C_4_/C_5_) in 4-O-5′ structures resulting during laccase (*T. villosa*) treatment of lignosulfonates, whereas Magina et al. [31] assigned the signal at δ_C_ 128 ppm to the C_5_ in 5-5′ structure formed during MtL laccase treatment of lignosulfonates. Therefore, these observations showed by ^13^C NMR also confirm the deprotonation of the lignin benzenic rings seen by HSQC and ^1^H NMR as a consequence of polymerization/condensation reactions by laccase enzymes, supporting the increment of molecular weight values (Section 3.2).

## 3. Materials and Methods

### 3.1. Raw Material, Laccases, and Chemicals

*Eucalypt (Eucalyptus globulus*) lignin was isolated by acid precipitation (pH 2.5) from Kraft black liquor, kindly supplied by La Montañanesa pulp mill (Lecta, Zaragoza, Spain). The chemical composition, determined by the Laboratory Analytical Procedures (LAP) [49], was as follows: acid-insoluble lignin (85.2 ± 0.3%), acid-soluble lignin (13.0 ± 0.1%), glucan (1.7 ± 0.1%), and xylan (1.8 ± 0.0%).

Two different laccases from bacterial and fungal origin were used: a recombinant bacterial laccase (SiLA) from *Streptomyces ipomoeae* CECT 3341.16 [50] and a commercial fungal laccase (MtL) from *Myceliophtora thermophila* (Novozym^®^ 51003), the latter kindly supplied by Novozymes (Bagsvaerd, Denmark). Enzyme activities (8 and 236,65 IU/mL for SiLA and MtL laccases, respectively) were estimated by oxidation of 5 mM 2,2′-azino-bis(3-ethylbenzothiazoline-6-sulphonic acid) (ABTS) to its cation radical (ε436 = 29 300 M^−1^ cm^−1^) in 0.1 mM sodium acetate (pH 5) at 24 °C.

All the reagents used were of analytical grade, purchased from either Sigma–Aldrich (Madrid, Spain) or Merck (Barcelona, Spain).

### 3.2. Kraft Lignin Laccase Reaction

A solution of 1.5 g/L of Kraft lignin was prepared in phosphate buffer at pH 7.0 and 8.0 (100 mM), according to optimal activity values for MtL and SiLA laccases, respectively [25,49]. Two different enzyme dosages and reaction times were used for both laccases (40 IU/g of lignin for 90 min and 100 IU/g of lignin for 240 min). The temperature was fixed at 45 and 60 °C, according to optimal activity values for SiLA and MtL laccases, respectively [25,50]. At the end of reactions, laccase-treated lignins were isolated by acid precipitation (pH 2.5), dried, and finally homogenized. Then, SiLA-KL1 and MtL-KL1 were referenced to Kraft lignins treated with SiLA and MtL laccases at lower enzyme dosage and reaction time, and SiLA-KL2 and MtL-KL2 to Kraft lignins treated at higher enzyme dosage and reaction time. 

### 3.3. Characterization of Kraft Lignin and Those Resulting from Laccase Reaction 

#### 3.3.1. Total Phenolic Content

The total phenolic content of lignin samples was determined according to Jiménez-López et al. [51]. Firstly, lignin samples were dissolved in dimethylsulfoxide (DMSO). Then, the absorbance at 760 nm of a mixture with 100 µL of lignin solutions, 500 µL of Folin–Ciocalteu reagent, and 400 µL of Na_2_CO_3_, was measured using a UV–Vis spectrophotometer (Lambda 365, PerkinElmer, Boston, MA, USA). A calibration curve prepared from a standard solution of gallic acid (1–200 mg L^−1^) was used to quantify the total phenolic content of lignin samples, which was expressed as mg gallic acid equivalent (GAE) g^−1^ of lignin (on a dry basis).

#### 3.3.2. Size Exclusion Chromatography (SEC)

SEC analysis (weight-average (Mw), number-average (Mn) molecular weights, and polydispersity (Mw/Mn)) were carried out in an Agilent Technologies 1260 HPLC (equipped with a G1315D DAD detector (Agilent, Waldbronn, Germany)) at 254 nm. Previously, lignin samples were dissolved at a final concentration of 0.5 g L^−1^ in NaOH (0.05 M). Two columns (Phenomenex, Torrance, CA, USA) coupled in series (GPC P4000 and P5000, both 300 × 7.8 mm) and a safeguard column (35 × 7.8 mm) were used. The operational conditions were described elsewhere [52].

#### 3.3.3. Fourier Transform Infrared Spectroscopy (FTIR)

FTIR spectra of lignin samples were obtained by a JASCO FT/IR 460 Plus spectrometer (Hachioji-shi, Tokyo), equipped with an accessory single reflection diamond, working with a resolution of 1 cm^−1^, 400 scans, and a spectral range of 2000–600 cm^−1^ [28].

#### 3.3.4. Nuclear Magnetic Resonance (NMR) 

Solid-state ^13^C nuclear magnetic resonance (^13^C NMR) analyses of lignin samples were carried out in a Bruker Avance III 400MHz (Billerica, MA, USA) at 100.64 MHz with the cross polarization/magic angle spinning (CP/MAS) technique. The operational conditions were described elsewhere [28]. Solution NMR spectra, including ^1^H NMR and heteronuclear single quantum correlation (HSQC) (2D NMR), were recorded on 40 mg of lignins (dissolved in 0.75 mL of deuterated dimethylsulfoxide (DMSO-d6)) in a Bruker AVANCE 500 MHz (Billerica, MA, USA), according to Eugenio et al. [28]. Residual DMSO (from DMSO-d6) was used as an internal reference (δ_C_/δ_H_ 39.6/2.5 ppm). Abundance of β-O-4′, resinols, spirodienones, arylglicerol, epiresinols, diaresinols, and Ar−CHOH−COOH substructures was estimated by 2D-NMR from Cα–Hα correlations. Cinnamyl alcohol end-groups using C_γ_–H_γ_ correlations, stilbenes (SB1 and SB5) using Cα–Hα correlations, C_2,6_–H_2,6_ correlations from S units, and C_2_–H_2_ correlations from G units were used to estimate the S/G lignin ratios.

## 4. Conclusions

Laccases oxidize phenolic lignin, leading to inter-unit linkages cleavage and, consequently, depolymerization. Simultaneously, the phenoxy radicals formed by the action of the laccase enzymes on the phenolic units, together with those derived from the cleavage of inter-unit linkages, undergo radical–radical coupling through phenyl ether-carbon and carbon-carbon links, resulting in new condensed structures. NMR characterization of eucalypt Kraft lignin treated by bacterial and fungal laccases provided structural changes that support both degradation and polymerization reactions. Compared to untreated lignin, HSQC analysis showed an almost complete absence of signals corresponding to lignin side-chains by laccase depolymerization. Simultaneously, a substantial diminution of aromatic signals was noticed by HSQC and ^1^H NMR after treatment with both laccases, which was attributed to polymerization by laccase reactions. Then, condensed structures, such as α-5′, 5-5′, and 4-O-5′, could be observed by HSQC and ^13^C NMR, supporting the increment in molecular weight and polydispersity values, as well as the reduction in the phenolic content, determined in lignins treated by both laccases.

## Figures and Tables

**Table 1:** Phenolic content, weight average (Mw) and number-average (Mn) molecular weights, and polidispersity (Mw/Mn) of untreated Kraft lignin and laccase-treated lignins. Mw and Mn are given in Da.

Samples	Phenolic Content (mg GAE/g Lignin)	Mw	Mn	D
Untreated Kraft lignin	552.4 ± 16.7	3530.5	525.5	6.7
SiLA-KL1	197.3 ± 8.2	6175.0	700.0	8.8
MtL-KL1	370.9 ± 20.0	6055.0	515.0	11.7
SiLA-KL2	189.2 ± 20.1	8375.0	715.0	11.7
MtL-KL2	339.0 ± 28.0	7835.5	435.5	18.0

SiLA-KL1 and MtL-KL1 for lignins treated with *S. ipomoeae* and *M. thermophila* laccases, respectively, at 40 IU/g of lignin for 90 min, and SiLA-KL2 and MtL-KL2 for lignins treated at 100 IU/g of lignin for 240 min.

## Data Availability

Not applicable.

## Supplementary Materials

The following supporting information can be downloaded at: https://www.mdpi.com/article/10.3390/ijms24032359/s1.
