# Supplementary material for: NMR Study on Laccase Polymerization of Kraft Lignin Using Different Enzymes Source
Source: Int J Mol Sci. 2023 Jan 25;24(3):2359. doi: 10.3390/ijms24032359 (PMC9917248; doi:10.3390/ijms24032359)
Supplement: Supplementary file 1 [file ijms-24-02359-s001.zip › ijms-2141442-supplementary.pdf]

International Journal of
Molecular Sciences

MDPI

Supplementary Material

NMR Study on Laccase Polymerization of Eucalypt Kraft Lignin Using Different Enzymes Source

David Ibarra[1*], Luisa García-Fuentevilla[1], Gabriela Domínguez[2], Raquel Martín-Sampedro[1], Manuel Hernández[2], María E. Arias[2], José, I. Santos[3], María E. Eugenio[1]

[1] Forest Sciences Institute (ICIFOR-INIA), CSIC, Ctra. de la Coruña Km 7.5, 28040 Madrid, Spain; ibarra.david@inia.csic.es (D.I.); luisa.garcia@inia.csic.es (L.G.-F.); raquel.martin@inia.csic.es (R.M.-S.); mariaeugenia@inia.csic.es (M.E.E.)

[2] Department of Biomedicine and Biotechnology. University of Alcalá. 28805 Alcalá de Henares, Madrid, Spain; gabriela.dominguez@edu.uah.es (G.D.); manuel.hernandez@uah.es (M.H.); enriqueta.arias@uah.es (M.E.A.)

[3] General Services of Research SGIKER, University of the Basque Country (UPV/EHU), Edificio Joxe Mari Korta Avda. Tolosa 72, Donostia-San Sebastian 20018, Spain; joseignacio.santosg@ehu.eus (J.I.S.)

* Correspondence: ibarra.david@inia.csic.es; Tel.: +34-913473948

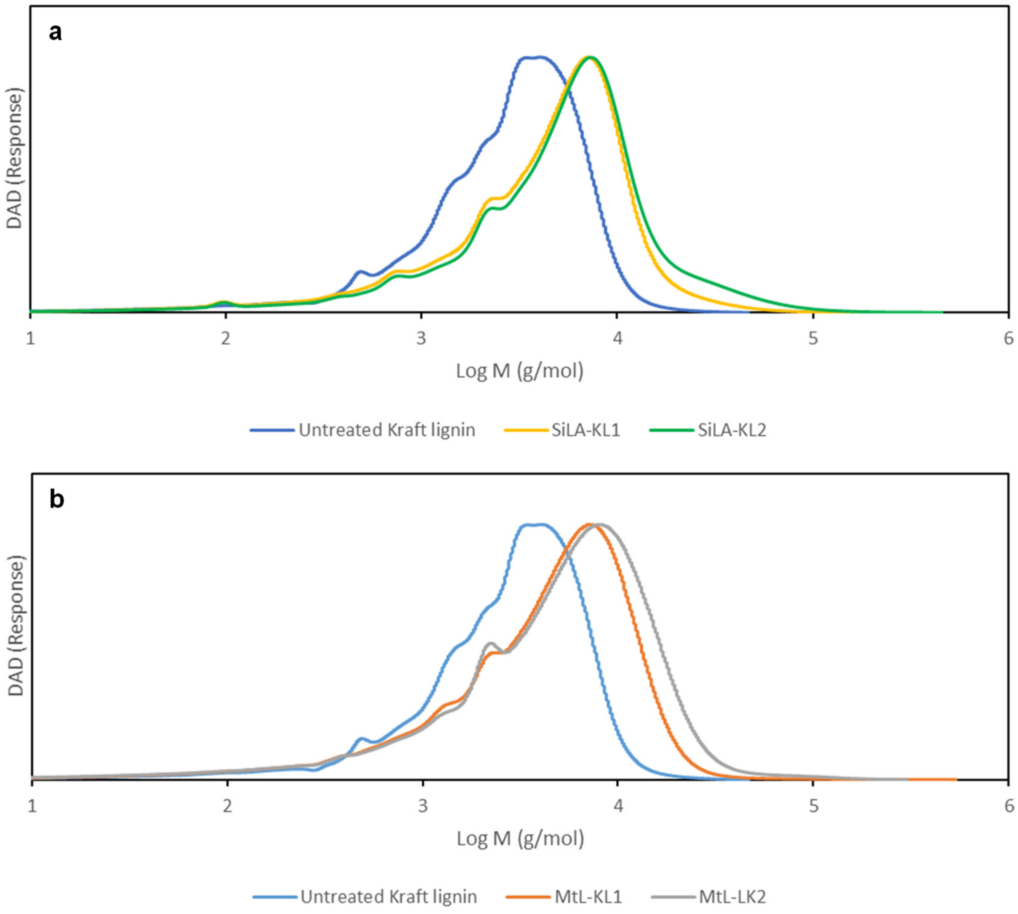

Figure S1. Molecular weight distributions of untreated Kraft lignin and the resulting treated lignins with SiLA (**a**) and MtL (**b**) laccases. SiLA-KL1 and MtL-KL1, 40 IU/g lignin and 90 min; SilA-KL2 and MtL-KL2, 100 IU/g lignin and 240 min.

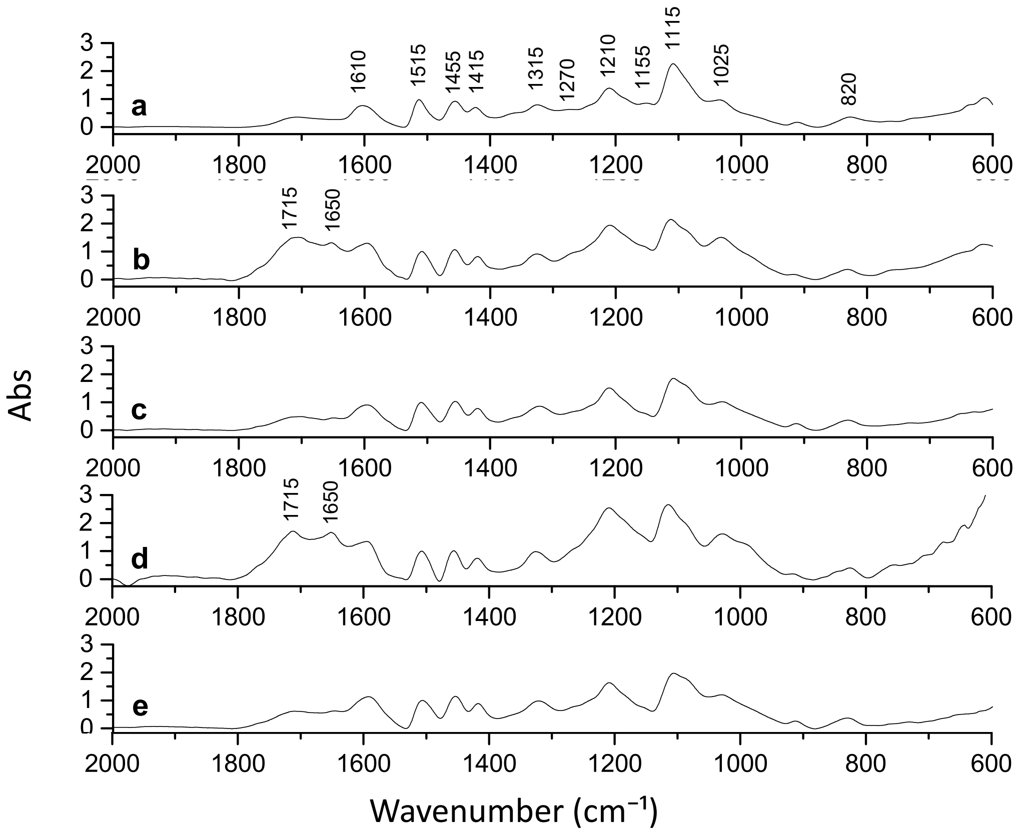

Figure S2. FTIR spectra, 2000-600 cm^{-1} region of untreated Kraft lignin (**a**) and the resulting treated lignins with SiLA (**b**, 40 IU/g lignin and 90 min, SiLA-KL1; **d**, 100 IU/g lignin and 240 min, SilA-KL2) and MtL (**c**, 40 IU/g lignin and 90 min, MtL-KL1; **e**, 100 IU/g lignin and 240 min, MtL-KL2) laccases. The bands in each spectra are normalized with regard to the band at 1515 cm^{-1}.

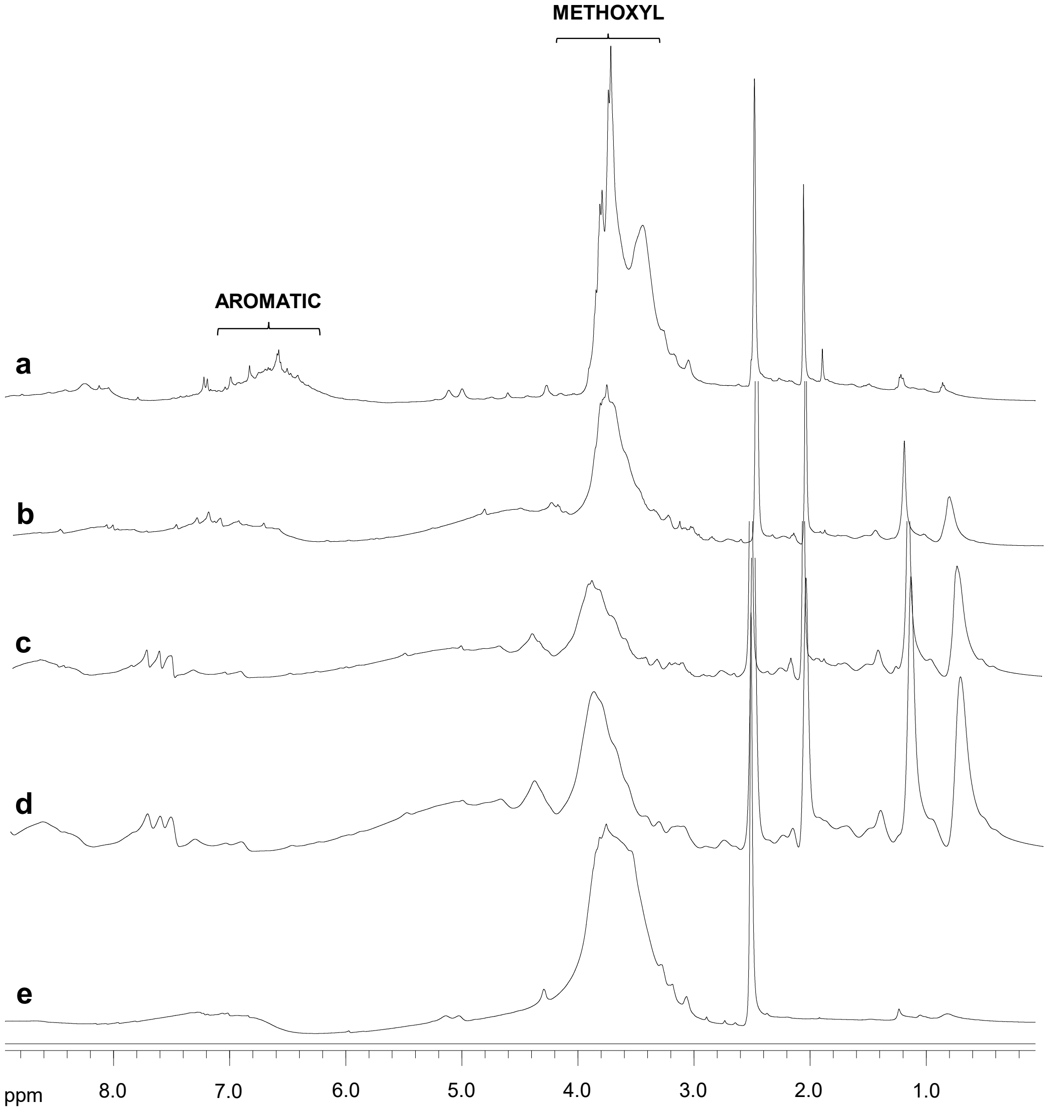

Figure S3. ^{1}H NMR spectra, δ_H 0.0–9.0 ppm, of untreated Kraft lignin (**a**) and the resulting treated lignins with SiLA (**b**, SiLA-KL1; d, SiLA-KL2) and MtL (**c**, MtL-KL1; **e**, MtL-KL2) laccases.

Table S1. Assignment of main lignin and carbohydrates $^{13}C–^{1}H$ correlation signals in the HSQC spectra of untreated Kraft lignin and the resulting treated lignins with MtL and SiLA laccases.

δ_C/δ_H (ppm)	Assignment
48.8/3.18	$C_\beta–H_\beta$, diaresinol substructures (**B′′**)
49.7/3.34	$C_\beta–H_\beta$, epiresinol substructures (**B′**)
51.8/3.4	$C_\alpha–H_\alpha$, α-5′ condensed substructure (**C**)
54.0/2.80	$C_\beta–H_\beta$, epiresinol substructures (**B′**)
53.8/3.04	$C_\beta–H_\beta$, resinol substructures (**B**)
56.0/3.71	C–H, methoxyls (**MeO**)
60.6/3.40–3.64	$C_\gamma–H_\gamma$, β-O-4′ substructures (**A**)
61.8/4.11	$C_\gamma–H_\gamma$, cinnamyl alcohol end groups (**I**)
63.4/3.23–3.87	$C_5–H_5$, xylan
63.6/3.10	$C_\gamma–H_\gamma$, aryl-glycerol (**AG**)
69.3/3.30–3.70	$C_\gamma–H_\gamma$, epiresinol substructures (**B′**)
70.1/3.73–4.10	$C_\gamma–H_\gamma$, epiresinol substructures (**B′**)
71.4/3.77–4.16	$C_\gamma–H_\gamma$, resinol substructures (**B**)
72.3/4.87	$C_\alpha–H_\alpha$, β-O-4′ **S** unit (**A**)
73.0/3.07	$C_2–H_2$, xylan
74.0/4.41	$C_\alpha–H_\alpha$, aryl-glycerol (**AG**)
74.3/3.31	$C_3–H_3$, xylan
74.3/4.43	$C_\alpha–H_\alpha$, Ar–CHOH–COOH units (**F**)
75.6/3.47	$C_\alpha–H_\alpha$ aryl-glycerol (**AG**)
75.9/3.51	$C_4–H_4$, xylan
81.6/4.75	$C_\alpha–H_\alpha$, spirodienone substructures (**E**)
81.8/4.76	$C_\alpha–H_\alpha$, epiresinol substructures (**B′**)
85.5/4.76	$C_{\alpha'}–H_{\alpha'}$, spirodienone substructures (**E**)
85.4/4.62	$C_\alpha–H_\alpha$, resinol substructures (**B**)
87.6/4.31	$C_\alpha–H_\alpha$, epiresinol substructures (**B′**)
101.9/4.30	C-1, (1-4) β-D-Xylp
104.1/6.60	$C_{2,6}–H_{2,6}$, S units (**S**)
103.9/6.82	$C_{2,6}–H_{2,6}$, 3,5-tetramethoxy-*para*-diphenol substructures ($\mathbf{S_{1\text{-}1'}}$)
105.0/6.9	$C_{2,6}–H_{2,6}$, $\mathbf{S_1\text{-}G_{1'}}$ / $\mathbf{G_{5'}}$ substructures
107.0/7.30	$C_{2,6}–H_{2,6}$, oxidized (H–C_α=O or $H_3C–C_\alpha$=O) S units (**S′**)
110.8/6.90	$C_2–H_2$, G units (**G**)
111.3/7.37	$C_2–H_2$, oxidized (H–C_α=O) G units (**G′**)
115.0/6.72	$C_{3,5}–H_{3,5}$, *p*-hydroxyphenyl (**H**)
115.1/6.40-6.80	$C_5–H_5$, G units (**G**)
119.6/6.76	$C_6–H_6$, G units (**G**)
119.7/6.95	$C_6–H_6$, 3-dimethoxy-*para*-diphenol substructures ($\mathbf{G_{1\text{-}1'}}$)
120.3/7.23	$C_\beta–H_\beta$, stilbene ($\mathbf{SB5_\beta}$)
123.4/7.50	$C_6–H_6$, oxidized ($H_3C–C_\alpha$=O) G units (**G′′**)
126.4/6.95	$C_\alpha–H_\alpha$, stilbene ($\mathbf{SB1_\alpha}$)
126.8/7.40	$C_6–H_6$, oxidized (H–C_α=O) G units (**G′**)
